# Diet-induced changes in the jejunal microbiota of developing broilers reduce the abundance of *Enterococcus hirae* and *Enterococcus faecium*

**DOI:** 10.1186/s12864-024-10496-8

**Published:** 2024-06-23

**Authors:** Paul B. Stege, Dirkjan Schokker, Frank Harders, Soumya K. Kar, Norbert Stockhofe, Vera Perricone, Johanna M. J. Rebel, Ingrid C. de Jong, Alex Bossers

**Affiliations:** 1 Wageningen Bioveterinary Research, Wageningen University and Research, Lelystad, Netherlands; 2 Wageningen Livestock Research, Wageningen University and Research, Wageningen, Netherlands; 3 Department of Veterinary Medicine and Animal Science, University of Milan, Milan, Italy

**Keywords:** Diet composition, Metagenomics, Butyrate supplementation, Poultry pathogens

## Abstract

Modern broiler breeds allow for high feed efficiency and rapid growth, which come at a cost of increased susceptibility to pathogens and disease. Broiler growth rate, feed efficiency, and health are affected by the composition of the gut microbiota, which in turn is influenced by diet. In this study, we therefore assessed how diet composition can affect the broiler jejunal gut microbiota. A total of 96 broiler chickens were divided into four diet groups: control, coated butyrate supplementation, medium-chain fatty acid supplementation, or a high-fibre low-protein content. Diet groups were sub-divided into age groups (4, 12 and 33 days of age) resulting in groups of 8 broilers per diet per age. The jejunum content was used for metagenomic shotgun sequencing to determine the microbiota taxonomic composition at species level. The composed diets resulted in a total of 104 differentially abundant bacterial species. Most notably were the butyrate-induced changes in the jejunal microbiota of broilers 4 days post-hatch, resulting in the reduced relative abundance of mainly *Enterococcus faecium* (-1.8 l2fc, P_adj_ = 9.9E-05) and the opportunistic pathogen *Enterococcus hirae* (-2.9 l2fc, P_adj_ = 2.7E-08), when compared to the control diet. This effect takes place during early broiler development, which is critical for broiler health, thus exemplifying the importance of how diet can influence the microbiota composition in relation to broiler health. Future studies should therefore elucidate how diet can be used to promote a beneficial microbiota in the early stages of broiler development.

**Supplementary Information:**

The online version contains supplementary material available at 10.1186/s12864-024-10496-8.

## Introduction

The continuous expansion of the poultry industry comes with the demand to improve sustainability of production. Modern broiler breeds offer the advantage of rapid growth and increased feed efficiency, but come with the disadvantage of increased susceptibility to physiological and metabolic disorders, and have indications of inferior immunity [1–6]. Although diet has also been optimized for sustainability in terms of growth rate and feed efficiency, the effect of diet on the composition of the intestinal microbiota has not been fully explored. Namely, the composition of the broiler jejunal microbiota and the potential diet-induced effects thereof are currently unknown, which is of specific interest as it is one of the principal sites of nutrient absorption [21].

The gastrointestinal tract and its associated microbiota jointly contribute to feed efficiency, the development of the immune system and ultimately to the state of health and disease [7–10]. In turn, diet composition is known to affect both intestinal physiology and microbiota composition, and is thus proposed as a tool to facilitate sustainability in terms of feed efficiency, animal health and reduced mortality. The relevance of intestinal microbiota alteration was first highlighted when growth promoters in the form of antibiotics were established to affect the microbiota and increase performance and feed efficiency of chickens [11–14]. However, this sub-therapeutic use of antibiotics has the added effect to enrich for antibiotic resistant bacteria, leading to its prohibition in regions such as Europe, the United States, and parts of Asia [15–21]. As a result, previous studies have investigated the impact of diet composition and additives on the intestinal microbiota in search for alternatives to mimic antibiotic-driven beneficial effects, such as enhanced performance and feed efficiency [22–24]. In these studies, 16S rRNA gene sequencing remains the most common approach to determine diet-induced effects in the bacterial community composition, but its resolution is surpassed by that of metagenomic shotgun sequencing (MSS). By sequencing the full microbiome, MSS is able to determine bacterial species and can be used to study gene composition and their corresponding gene pathways [25].

The small intestine is specialized for nutrient absorption, where medium-chain fatty acids are mainly absorbed in the proximal part of the small intestine, amino acids in the proximal part of the jejunum, and long-chain fatty acids in the distal parts of the jejunum [26–29]. The small intestine is densely colonized with bacteria and in the case of broiler chickens, the most abundant bacteria include lactic acid-producing bacteria *Lactobacillus, Enterococcus* and *Streptococcus*, from which *Lactobacillus* is overall the most abundant genera [30–34]. The high abundance of *Lactobacillus* suggests that these bacteria play a prominent role in the intestine and is one of the reasons why *Lactobacillus* is commonly applied as chicken probiotic [35, 36]. Diet composition is explored as an approach to induce shifts in the intestinal microbiota, for instance by altering the ratio of fatty acids and fibres in feed. When animal fat and soybean oil were supplemented with medium-chain fatty acids (MCFAs; 0.3% C10 and 2.7% C12) for 34 days, the broiler ileum microbiota showed a reduction of *Lactobacillus*, Enterococcaceae, Micrococcaceae and an increase in Enterobacteriaceae [22]. MCFAs have been observed to have antibacterial properties against opportunistic pathogens like *Clostridium perfringens* and *Escherichia coli* when applied in in vitro experiments, but it is unknown if the antibacterial properties persist in a complex system as the intestinal microbiota [37–39]. Another example is butyrate, which is a short-chain fatty acid (SCFA) and is the preferred energy-providing substrate of colonocytes [40]. When broiler feed was supplemented with butyrate for 42 days, both feed efficiency and villi size were increased [23]. Butyrate can be rapidly absorbed by the microbiota and intestinal cells located in the proximal sites of the intestine. In order to slowly release butyrate over the full length of the intestine, Mallo et al., 2021, supplemented coated butyrate for 42 days and observed similar results to uncoated butyrate [41]. Supplementation of fibre in feed is known to induce changes in the intestinal microbiota of broilers. Mainly the bacteria located in the caecal microbiota can ferment fibre, generating components including SCFAs [42]. While low level fibre supplementation can increase the amount of butyric acid in the cecum of 21-day-old broilers and increased the abundance of *Helicobacter pullorum* and *Megamonas hypermegale*, high levels of fibre supplementation increased the abundance of taxa that may include pathogens, namely Selenomonadales, Enterobacteriales, and Campylobacterales [24]. Qiuyu J. et al., 2024 also observed that fibre supplementation in 21 day old broilers resulted in the increase in *Escherichia*-*Shigella* (i.e. Enterobacteriales), but additionally observed an increase of *Bifidobacterium* and *Lactobacillus* [43]. The genera of *Bifidobacterium* and *Lactobacillus* may include species that are considered beneficial and are applied in probiotics [44].

The majority of previously discussed studies analyse diet-induced effects on the genera taxonomic-level of bacteria, preventing the observation of species-specific effects. This lack of resolution can result in the neglection of important bacteria, including pathogens. Moreover, the effect of diet on the jejunal microbiota are unknown, while it is one of the principal sites of nutrient absorption. In this study, we therefore assessed how different diets impact the composition of the jejunal microbiota on the species level by performing MSS on broilers at 4, 12 and 33 days post-hatch.

## Results

### Jejunal microbiota composition across diet groups

**Fig. 1:**
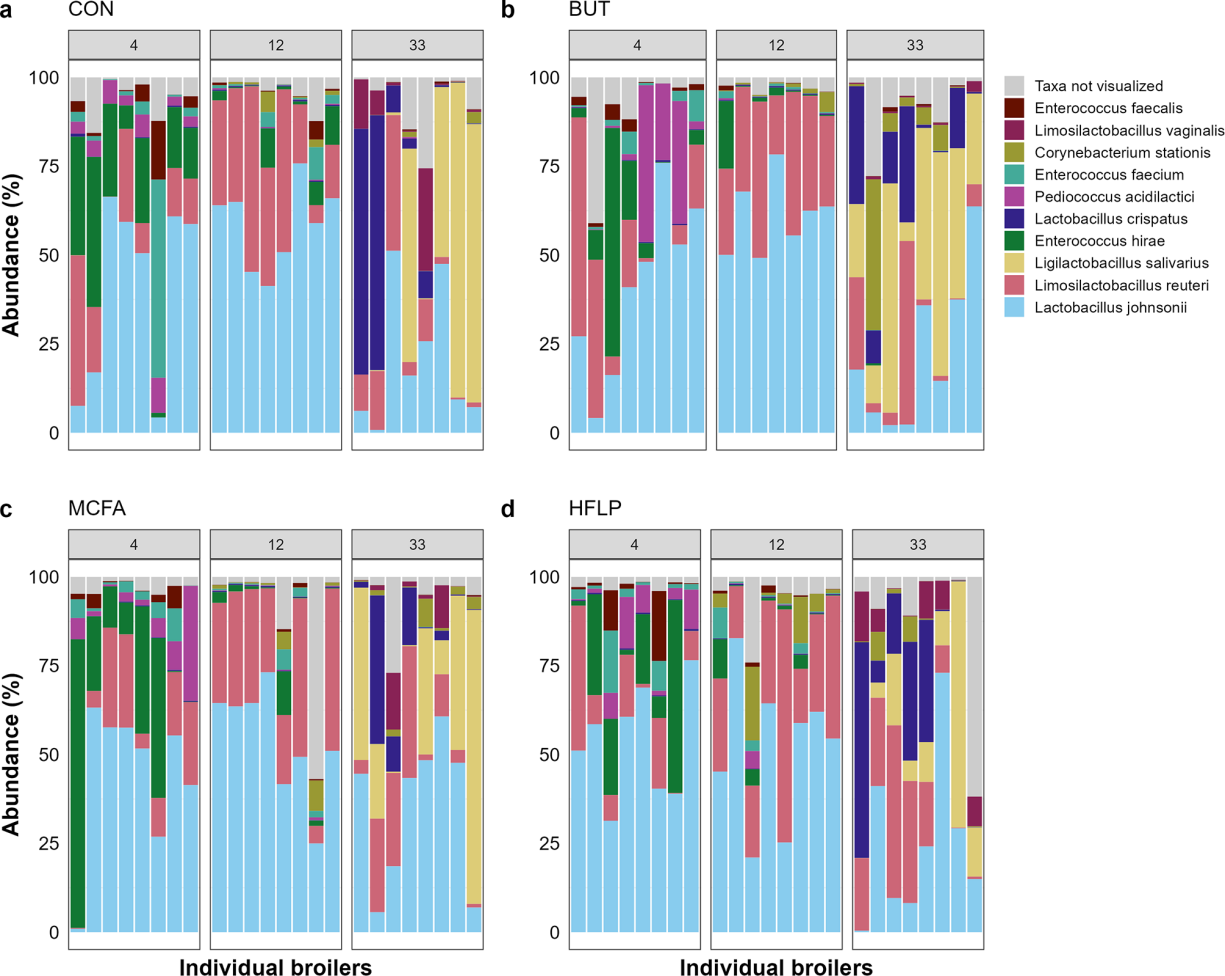
Jejunal microbiota composition per diet group at 4, 12 and 33 days post-hatch. Relative abundance of the 10 most abundant bacterial species per diet group. (**a**) Control diet (CON), (**b**) control diet plus butyrate (BUT), (**c**) control diet plus medium-chain fatty acids (MCFA) and (**d**) a high-fibre low-protein diet (HFLP). Broilers are grouped by columns, representing the number of days post-hatch. Abundance was plotted on the relative abundance scale from 1 to 100%. Each colour represents a different species (see legend)

A total of 96 Ross 308 broilers were housed in floor pens. They were divided into four diet groups, to study the effect of diet: (1) control diet (CON); (2) control diet supplemented with butyrate (BUT), (3) control diet supplemented with medium-chain fatty acids (MCFA) and (4) a diet with high-fibre low-protein composition (HFLP). The jejunal microbiota was studied by taking jejunal content samples after either 4, 12 or 33 days post-hatch, thus studying groups of 8 broilers per diet per timepoint. Samples were used for metagenomic shotgun sequencing, resulting in 137.6 M [SEM 67.4] reads per sample and 32.7 M [SEM 2.5] assigned read pairs per sample after taxonomic classification. Sample s2229 contained the lowest number of assigned read pairs (1.7 M) and was therefore excluded from downstream analysis. This sample was part of the BUT group 12 days post-hatch. The top 10 most abundant species was consistent across all diet groups, comprising the following 10 species: *Lactobacillus johnsonnii*, *Limosilactobacillus reuteri*, *Ligilactobacillus salivarius*, *Enterococcus hirae*, *Lactobacillus crispatus*, *Pediococcus acidilactici*, *Enterococcus faecium*, *Corynebacterium stationis*, *Limosilactobacillus vaginalis* and *Enterococcus faecalis* (Fig. 1). These are all lactic acid bacteria, except for *C. stationis* [45, 46]. The jejunal microbiota displays a significant age-dependent effect, independent of diet, as revealed by Principal Coordinate Analysis (PCoA) of Bray-Curtis dissimilarity matrices (Pval = 0.001, figure s1). Principle Response Curve (PRC) analysis was used to highlight five species that show the largest change in relative abundance over time, accounting for the influence of different diet groups, compared to the control group (figure s2). This revealed the overall high abundances of *L*. *johnsonnii* and *L. reuteri* at 12 days post-hatch (58.99% [SEM 0.03], 28.89% [SEM 0.02]) compared to 33 days post-hatch (18.16% [SEM 0.04], 6.99% [SEM 0.03]). In contrast, *L. salivarius* and *L. crispatus* showed low abundances at 12 days post-hatch (0% [SEM 0.00]; 0.31% [SEM 0.00]) but high abundances at 33 days post-hatch (20.05% [SEM 0.28]; 6.86% [SEM 0.04]). *C*. *stationis* was present in low abundance at 12 days post-hatch (0.83% [SEM 0.01]) and in slightly lower abundance at 33 days post-hatch (0.67% [SEM 0.01]).

**Fig. 2:**
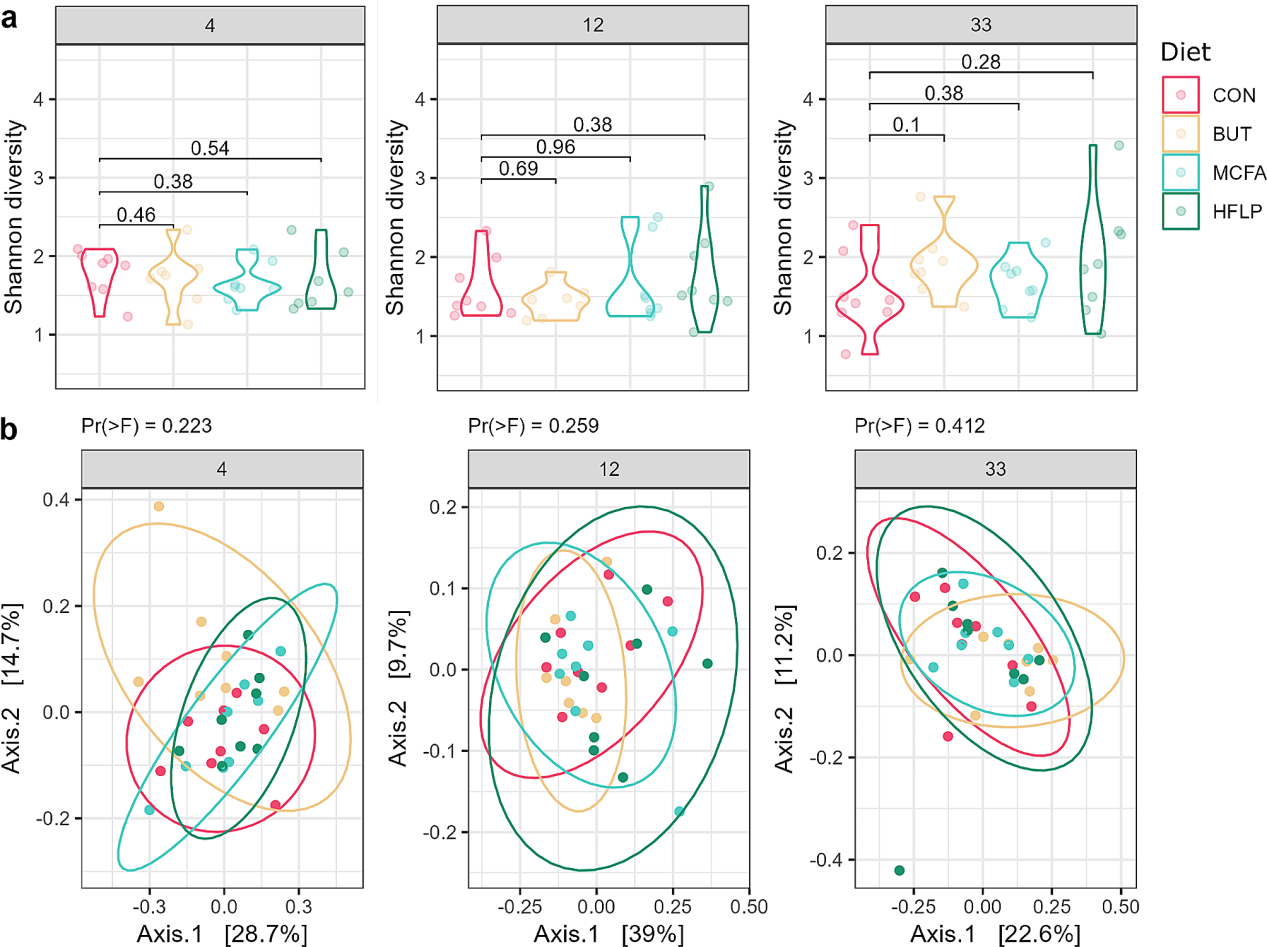
Diversity indices of jejunal microbiota per diet group at 4, 12 and 33 days post-hatch. (**a**) Alpha diversity per diet group expressed by Shannon diversity index on OTU level. Diet groups did not differ in terms of alpha diversity when compared with Wilcoxon rank-sum tests. (**b**) Beta diversity of bacterial species, using principal coordinate analysis (PCoA) of Bray-Curtis dissimilarity on OTU level. Individual broilers and corresponding ellipses are coloured according to diet group (see legend). Plot panels represent broiler groups of 4, 12 and 33 days post-hatch. Permutational multivariate analysis of variance (PERMANOVA) and testing for homogeneity of multivariate dispersions revealed no significant differences between groups

The jejunal microbiota diversity expressed as Shannon index and the microbiota evenness expressed as Pielou index, were not significantly different between diet groups (Fig. 2a, figure s3). Overall, the total species diversity is highly similar among diet groups. PCoA of Bray-Curtis dissimilarity matrices revealed that diet was not a main driver of the observed variance in microbiota composition between samples at either 4, 12 or 33 days post-hatch (Fig. 2b).

**Fig. 3:**
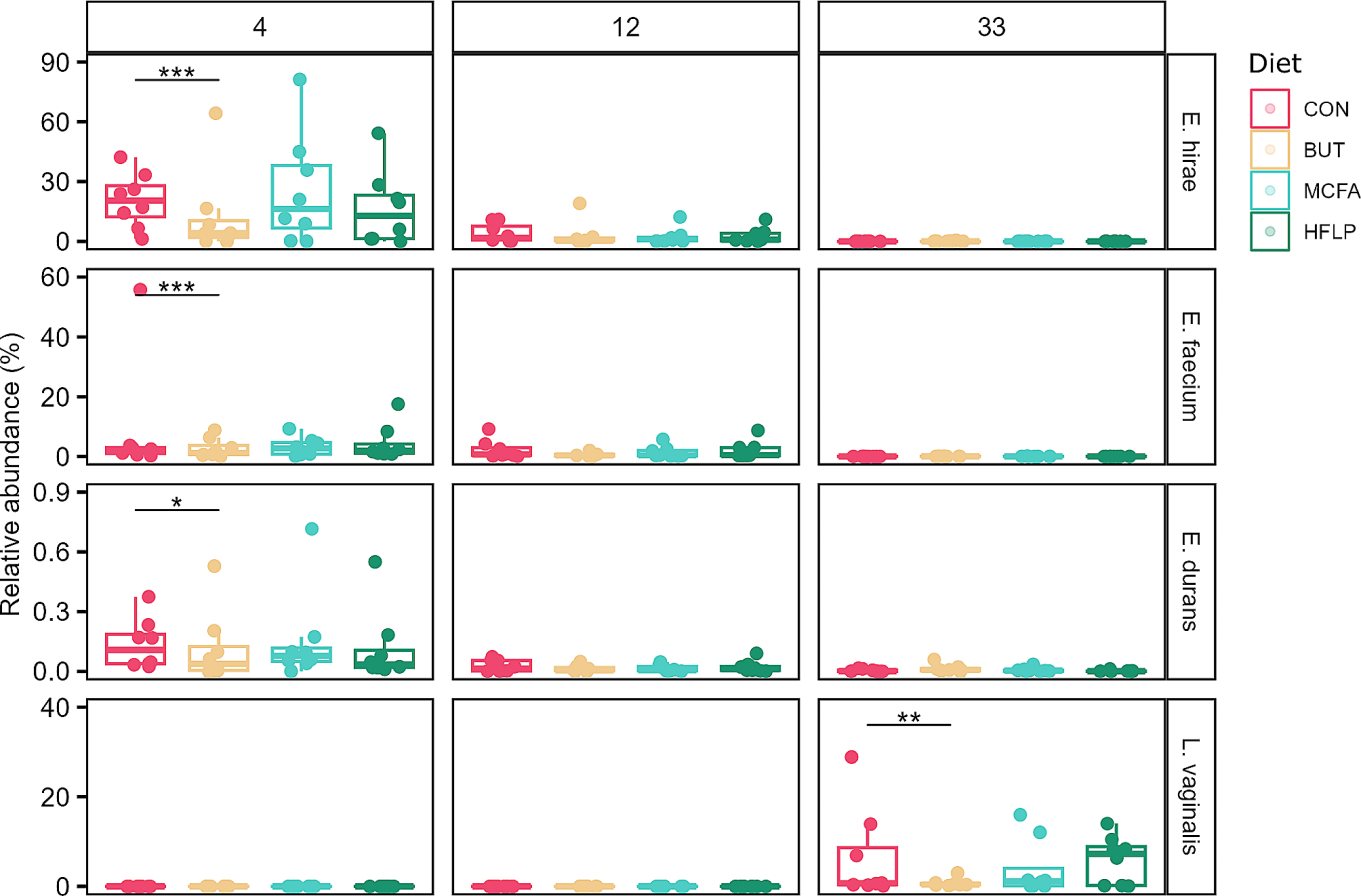
Differentially abundant confirmed bacterial species per diet group at 4, 12, 33 days post-hatch. Rows indicate the relative abundance of opportunistic pathogens *E. hirae*, *E*. *faecium*, *E*. *durans* and potential beneficial bacteria *L*. *vaginalis*. Columns represent broiler groups of 4, 12 and 33 days post-hatch. Individual broilers are coloured according to diet group (see legend). Adjusted *p*-values are calculated as part of ANCOMBC as function of the control diet and indicated by *< 0.05, **< 0.01 and ***< 0.001

Differential abundance analysis revealed a total of 104 bacterial species that were significantly different in terms of abundance when comparing diet groups to the control group (Fig. 3, table s1-s9). At 4 days post-hatch, the comparison of the jejunal microbiota of the **BUT** diet group to the control group resulted in 43 differentially abundant bacteria. Bacteria with a relative abundance above 0.01% and that changed in terms of relative abundance compared to the control group (*p*-value < 0.05), expressed as log2 fold changes (l2fc), included: a reduction of *E. hirae* (-2.9 l2fc, 4.2% abundance), *Enterococcus faecium* (-1.8 l2fc, 1.2% abundance), *Enterococcus durans* (-2.6 l2fc, 0.04% abundance), *Erysipelatoclostridium ramosum* (-2.4 l2fc, 0.03% abundance), *Enterococcus avium* (-1.8 l2fc, 0.02% abundance), *Lacrimispora saccharolytica* (-1.5 l2fc, 0.01% abundance), *Massilistercora timonensis* (-1.5 l2fc, 0.01% abundance), *Lachnoclostridium phocaeense* (-1.4 l2fc, 8.3e-3% abundance) and of *Weissella paramesenteroides* (-5.1 l2fc, 2.2e-4% abundance). Comparing the **MCFA** diet group to the control group resulted in six differentially abundant bacteria that were present in low abundance; including a reduction of *Pediococcus pentosaceus* (-1.6 l2fc, 0.04% abundance) and of *W*. *paramesenteroides* (-4.8 l2fc, 2.8e-4% abundance). Comparing the **HFLP** diet group to the control group, resulted in seven differentially abundant bacteria that were present in low abundance, including a reduction of *P*. *pentosaceus* (-1.3 l2fc, 0.043% abundance) and of *W*. *paramesenteroides* (-5.1 l2fc, 7.3e-5% abundance).

At 12 days post-hatch, the comparison of the **BUT** diet group to the control group revealed 17 differentially abundant bacteria that were present in low abundance, including a reduction of *L. brevis* (-2.1 l2fc, 1.1e-3% abundance). The **MCFA** diet group and control group comparison revealed three differentially abundant bacteria that had a low relative abundance in both groups (all below 1e-2% abundance, table s5). Comparing the **HFLP** diet group to the control group revealed 31 differentially abundant bacteria, including the increase of *Staphylococcus pseudoxylosus* (1.7 l2fc, 0.71% abundance), *Corynebacterium ammoniagenes* (2.1 l2fc, 0.03% abundance), *Arthrobacter* sp. YC-RL1 (4.2 l2fc, 0.018% abundance and the decrease of *W. paramesenteroides* (-3.6 l2fc, 4.2e-4% abundance), *Limosilactobacillus mucosae* (-2.2 l2fc, 9.7e-5% abundance) and *L. brevis* (-2.3 l2fc, 1.4e-3% abundance).

At 33 days post-hatch, the **BUT** diet group and control group comparison revealed 18 differentially abundant bacteria, including a reduction of *L. vaginalis* (-2.5 l2fc, 0.42% abundance), *Lactobacillus amylovorus* (-2.2 l2fc, 0.07% abundance, *Lactobacillus helveticus* (-2.3 l2fc, 0.05% abundance), *P. acidilactici* (-1.2 l2fc, 0.01% abundance) and the increase of *Corynebacterium casei* (1.1 l2fc, 0.26% abundance) and *Jeotgalicoccus* (*Micrococcus*) *candicans* (1.5 l2fc, 0.03% abundance). The **MCFA** diet group and control group comparison revealed 33 differentially abundant bacteria, including the increased abundance of *Corynebacterium stationis* (2.3 l2fc, 1.7% abundance), *C*. *casei* (1.8 l2fc, 0.16% abundance), *Corynebacterium glutamicum* (1.3 l2fc, 0.12% abundance), *Aerococcus urinaeequi* (1.6 l2fc, 0.04% abundance), *J.* (*M.*) *candicans* (2.3 l2fc, 0.02% abundance), *C*. *ammoniagenes* (2.1 l2fc, 0.02% abundance) and *Corynebacterium deserti* (1.8 l2fc, 0.01% abundance). Comparing the **HFLP** diet group to the control group revealed 23 differentially abundant bacteria were present in low abundance in both groups (all below 1e-2% abundance, table s9).

### Confirmation of opportunistic pathogenic and potential beneficial bacterial species

The observed differentially abundant bacteria included the opportunistic pathogens *E*. *hirae*, *E*. *faecium*, *E*. *durans* and *S. pseudoxylosus*. From this selection, *E*. *hirae*, *E*. *faecium* and *E*. *durans* are present in high relative abundance in broilers in the control group at an early stage of broiler development (20.53% [SEM 0.14], 1.86% [SEM 0.19], 0.11% [SEM 0.00]) at 4 days post-hatch compared to broilers of 12 days post-hatch (1.75% [SEM 0.05], 0.66% [SEM 0.03], 0.01% [SEM 0.00], Fig. 3). *E*. *faecium*, *E*. *durans* and *E*. *hirae* were all shown to decrease in abundance at 4 days post-hatch as a result of butyrate supplementation compared to the control diet. In order to confirm the presence of these closely related species and exclude the possibility of incorrect annotation by the aligner tool, sequencing data of the control diet group 4 days post-hatch was directly mapped to the genomes of all detected enterococcal species (105.1 M [SEM 33.0] reads per sample). This resulted in a high number of reads per sample mapping to *E*. *hirae* (10.0 M [SEM 6.0] reads, 90.9% [SEM 5.3] coverage, 415.7 [SEM 286.1] depth), *E*. *faecium* (0.8 M [SEM 0.3] reads, 88.0% [SEM 2.1] coverage, 31.4 [SEM 15.8] depth), *E*. *faecalis* (0.3 M [SEM 0.3] reads, 88.6% [SEM 6.6] coverage, 12.4 [SEM 14.6] depth) and in lesser extend to other enterococcal species (all below 70% coverage), thus confirming that *E*. *hirae* and *E*. *faecium* are present in the jejunal microbiota of these broilers at 4 days post-hatch (table s10). This analysis was repeated for the bacteria *L*. *mucosae*, *L*. *vaginalis*, *L*. *brevis*, *L*. *amylovarus, L*. *helveticus*, *P*. *pentosaceus* and *W*. *paramesenteroides* (supplemental data, table s10). From these bacteria, only the presence of *L*. *vaginalis* could be confirmed at 33 days post-hatch (0.4 M [SEM 2.2] reads, 86.8% [SEM 10.9] coverage and 26.2 [SEM 140.5] depth). *L*. *vaginalis* was therefore confirmed to be present at 0.71% [SEM 0.04] relative abundance in the control group at 33 days post-hatch (Fig. 3).

## Discussion

In this study, we determined the jejunal bacterial microbiota of broilers 4, 12 and 33 days post-hatch using metagenomic shotgun sequencing (MSS) to evaluate to what extend diet can modulate the jejunal microbiota composition. The results reveal that diet supplementation with either butyrate (BUT), medium-chain fatty acids (MCFA) or diet with high-fibre low-protein content (HFLP), can induce significant differences in the relative abundance of a total of 104 bacterial species. The results of butyrate supplementation are of specific interest, since supplementation reduced the relative abundance of highly abundant enterococci in the jejunal microbiota 4 days post-hatch; A critical stage for broiler health [47, 48]. Specifically, MSS allowed to differentiate between bacteria on species level and revealed that butyrate supplementation greatly reduces the relative abundance of both *Enterococcus hirae* and *Enterococcus faecium*.

Regardless of the fluctuations in microbiota composition in the first weeks of life, we observed that the most abundant species are lactobacilli. This includes the genera *Lactobacillus*, but also related genera such as *Limosilactobacillus*, as a result of the reclassification of *Lactobacillus* into 25 genera in 2020 [49]. The high abundance of lactobacilli is therefore in concordance with previous studies that analysed the small intestines of broilers [30, 33, 50]. MSS allowed us to surpass the taxonomic resolution of 16S rRNA gene sequencing. To our knowledge, this is the first time that the composition of broiler jejunal microbiota has been determined at the bacterial taxonomic species level, unbiased by 16S rRNA hyper-variable region amplification choices. The jejunal microbiota displayed an overall age-dependent effect, independent of diet. Principle Response Curve analysis revealed five species that show the largest change in abundance over time, while accounting for the influence of different diet groups compared to the control group. These species suggest a transition from highly abundant *Lactobacillus johnsonii* and *L*. *reuteri* (respectively, 58.99% [SEM 0.15] and 28.89% [SEM 0.02] relative abundance) at 12 days post-hatch to *Ligilactobacillus salivarius* and *Lactobacillus crispatus* (respectively, 20.35% [SEM 0.28] and 6.86% [SEM 0.21]) at 33 days post-hatch. This is similar to the findings of Lu et al., 2023, when studying the broiler ileum microbiota using 16S rRNA gene clone libraries. They observed a transition of the most dominant species, switching from *Lactobacillus acidophilus* at 14 days post-hatch (53% abundance) to *L. crispatus* at 28 days post-hatch (75% abundance) [30]. The ileum is the small intestinal region located directly downstream of the jejunum and the microbiota of these regions share similarities in their composition, potentially explaining these similar findings [51].

We observed *E. hirae* to be the second most abundant bacterial species in the jejunal microbiota 4 days post-hatch among diet groups (12.95% [SEM 0.21]) and observed a much lower relative abundance of *E*. *hirae* 12 days post-hatch (0.83% [SEM 0.05]). This is in line with the findings of Schokker et al., 2017, where a decrease in overall enterococcal abundance was observed from 21.7% 4 days post-hatch to 4.9% 14 days post-hatch [50]. When specifically comparing for differences in the microbiota between diet groups, we determined that supplementation of butyrate to broiler feed resulted in a 2.9 log2 fold change decrease in abundance of *E*. *hirae* in broilers 4 days post-hatch when compared to the control diet group. In addition, the butyrate supplemented group showed a decrease of several enterococci species at 4 days post-hatch, including *E. faecium* and *Enterococcus durans* and *Enterococcus avium*. From these, only *E*. *hirae* and *E*. *faecium* were present in sufficient abundance to ensure that this species is present with at least 70% genome coverage, leading us to conclude that butyrate supplementation induced a reduction in the relative abundance of *E. faecium* and *E*. *hirae*. To our knowledge, this is the first time this function is demonstrated in broilers and with sufficient resolution to distinguish between closely related enterococci species. The supplemented butyrate in this study is coated, which was previously found to result in the slow release of butyrate along the length of the intestinal tract [52, 53]. The effect of coated butyrate on enterococci is similar to the findings of Sun et al., 2022, where coated butyrate was shown to reduce the abundance of enterococci in the ileum microbiota of squabs [54]. *E*. *hirae* is an opportunistic pathogen that can cause locomotion problems, endocarditis and septicaemia in broilers [55–58]. The observed reduction in the abundance of *E*. *hirae* 4 days post-hatch is of specific interest, since this early stage represents the most critical period during broiler development. In this stage, the immune and digestive system are still immature, thereby increasing the susceptibility to disease [47]. This period is furthermore marked by the transition from aerial breathing, initiation of thermal regulation and changes in diet composition, from yolk to solid feed, contributing to the overall high stress load during early broiler development [48]. While some short-chain fatty acids directly inhibit bacterial growth, butyrate supplementation only resulted in limited growth inhibition of *E*. *faecium* and *E*. *hirae*, when tested in vitro [37–39, 59]. These in vitro results therefore suggest that the reduced abundance of *E*. *hirae* and *E*. *faecium* are not likely to be caused by butyrate directly, but rather indirect, i.e., by changes of the jejunal microbiota as a result of the butyrate supplementation.

Depending on the genetic makeup, *E*. *faecium* can act like an opportunistic pathogen or gut commensal [60–63]. Moreover, specific isolates of *E*. *faecium* are applied as broiler probiotics [64, 65]. The detected *E*. *faecium* genome should therefore first be determined in order to conclude about its pathogenic potential and impact on broiler health. In addition, we observed a reduced abundance of *L*. *vaginalis* as a result of butyrate supplementation 33 days post-hatch. In contrast to *E*. *hirae*, *L*. *vaginalis* is expected to be beneficial for gut health. These findings therefore suggest that that butyrate supplementation has a positive effect on the broiler jejunal microbiota 4 days post-hatch, but not at 33 days post-hatch [66, 67]. While this concerns the positive effects on the microbiota, previous studies have shown that the mainly positive effects of butyrate on broiler performance take place when supplemented for the duration of the starter phase (until 14–21 days) [68, 69]. While some studies confirm that this holds true for improved effects on broiler intestinal development as well, these results are ambiguous [52, 68, 70, 71]. Our results indicate potential negative effects of butyrate supplementation in broiler at 33 days post-hatch, which seems to be in line with the findings of our previous study, where butyrate supplementation was found to increase both Gram-negative bacteria abundance and endotoxin excretion in the cloacal microbiota at 35 days post-hatch [72]. Such information is essential to create ‘customized’ nutritional approaches specific to each production phase, with the goal of cultivating a favourable microbiota in broiler chickens. Additional differentially abundant bacteria included the potential pathogens *E*. *durans* and *S*. *pseudoxylosus* and the following bacteria that are used in probiotics since they are considered beneficial to gut health: *L*. *brevis*, *L*. *amylovarus*, *L*. *helveticus*, *P*. *pentosaceus* and *W*. *paramesenteroides* [44, 73–78]. There was, however, insufficient sequencing data to cover the genome of these species. Future studies should therefore validate whether the observed diet-induced effects concern these species or closely related species.

## Conclusions

Metagenomic shotgun sequencing allowed us to surpass analytic limitations on a genera taxonomic-level of bacteria and instead study species-specific effects. BUT, MCFA and HFLP diets induced changes in the jejunal microbiota composition at bacterial species level of broilers 4, 12 and 33 days post-hatch. Most notable was the effect of butyrate supplementation 4 days post-hatch, reducing the abundance of *E*. *faecium* and the opportunistic pathogen *E*. *hirae*. This early stage is critical for broiler health, emphasizing the role of diet in shaping the microbiota and its relation to broiler development and health. Future studies should elucidate how diets promote beneficial microbiota while suppressing additional pathogens like *Campylobacter* species, *Salmonella enterica*, *Escherichia coli*, and *Clostridium perfringens*. The incorporation of functional metagenomics and metatranscriptomics can additionally determine the role of understudied bacteria and reveal microbial activity changes linked to diet.

## Methods

### Classification of broiler groups

Day-old Ross 308 male broiler chickens were obtained from a commercial hatchery (Probroed & Sloot, Groenlo, The Netherlands), with an average weight of 43.3 g. They were housed in floor pens with wood shavings as substrate *ad libitum* access to feed and water as described by Perricone et al., 2023 [72]. In summary, a total of 1344 broilers were randomly allotted to one of six diets in a completely randomized block design. Broilers were kept in pens measured 1.10 × 1.90 m containing wood shavings and a perch. In order to prevent the exchange of manure and or litter, pens were separated by plywood panels. The temperature was set at 34 °C on day 0 and was gradually decreased to 20 °C over the course of 35 days [72]. All 1344 broilers were sampled for Perricone et al., while a subset of samples were selected for this project, in order to determine associations between the microbiota composition and the following four diets: (1) control diet without any supplementation (CON); (2) control diet supplemented with micro-encapsulated sodium butyrate (BUT, Excential Butycoat®, Orffa, Werkendam, the Netherlands), (3) control diet supplemented with a mixture of medium-chain fatty acids (MCFA, Aromabiotic®, Nuscience, Belgium) and (4) a diet with a higher fibre and lower protein content compared to the control diet (HFLP, table s11) [72]. While the BUT and MCFA diet involve supplementation of components, the HFLP diet involved substitutions of several components of the control diet, including the substitution of rapeseed meal by potato protein and an increase of sunflower seed meal and corn, and a reduction in soybean meal. To summarize, the described sample subset results into studying 8 broilers per diet per timepoint. Feed was provided *ad libitum* via a round feeder (diameter: 35 cm) hanging in the pen. Water was provided via seven nipples along the side wall of a pen. Broilers were vaccinated against infectious bronchitis before arrival at the experimental facility and on day 25, and against Newcastle disease at day 15.

### Sample collection, storage and DNA extraction

Jejunal chyme samples were taken at either 4, 12 or 33 days post-hatch. Broilers were first anaesthetized with Zoletil® and then euthanized via cervical dislocation. The jejunum was then isolated by excising a 10 cm segment, commencing from the Meckel’s diverticulum. The distal end of the segment was precisely aligned with the Meckel’s diverticulum and the diverticulum was included in the extraction. The jejunum content was subsequently squeezed into collection tubes, snap-frozen in liquid nitrogen and transferred to storage at -80 °C. One freeze-thaw cycle was introduced when dividing samples into aliquots of 0.2 g. Aliquoted samples were used for DNA extraction with the PureLink Genomic DNA Mini Kit (Invitrogen, Carlsbad, USA) according to the manufacturer’s instructions. Total DNA was quantified by using a 2200 Tapestation (Agilent, Santa Clara, USA).

### Metagenomic shotgun sequencing and data processing

DNA samples were sent to GenomeScan B.V. (Leiden, the Netherlands) for Metagenomic shotgun sequencing. Library preparation was performed using the NEBNext® Ultra II FS DNA module (E7810S, NEB, Ipswich, USA) and the NEBNext® Ultra II Ligation module (E7595S, NEB, Ipswich, USA) according to manufacturer’s protocols. Libraries were sequenced on a NovaSeq 6000 sequencer (Illumina, San Diego, USA) using S2 flow cells and the 2 × 150 bp paired-end kit (Illumina, San Diego, USA) according to company protocols. Samples contained on average 137.6 M [SEM 67.4] reads per sample. Sequencing reads were adapter-clipped, erroneous-tile filtered, and quality-trimmed at ≥ Q20 (PHRED score) using Bbduk v38.96 and subsequently filtered for host DNA using the global-alignment algorithm of BBmap v38.96 with default settings and *fast = t* (broiler genome version 2021/01/19, accession number GCF_016699485.2) [79]. Read pairs were then used for taxonomic classification by Kraken v2.1.2 using the premade standard Kraken RefSeq nucleotide database and applying a confidence cut-off of 0.3 (database version 5/17/2021) [80]. This resulted into 32.7 M [SEM 2.5] assigned read pairs per sample. The sample with the lowest number of assigned read pairs (1.7 M) was excluded from downstream analysis (s2229, BUT group 12 days post-hatch). Kraken2 read counts were exported using kraken-biom v1.0.1 with default settings *–min S –max O*, here referred to as OTU level [81].

### Data analysis

**Fig. 4:**
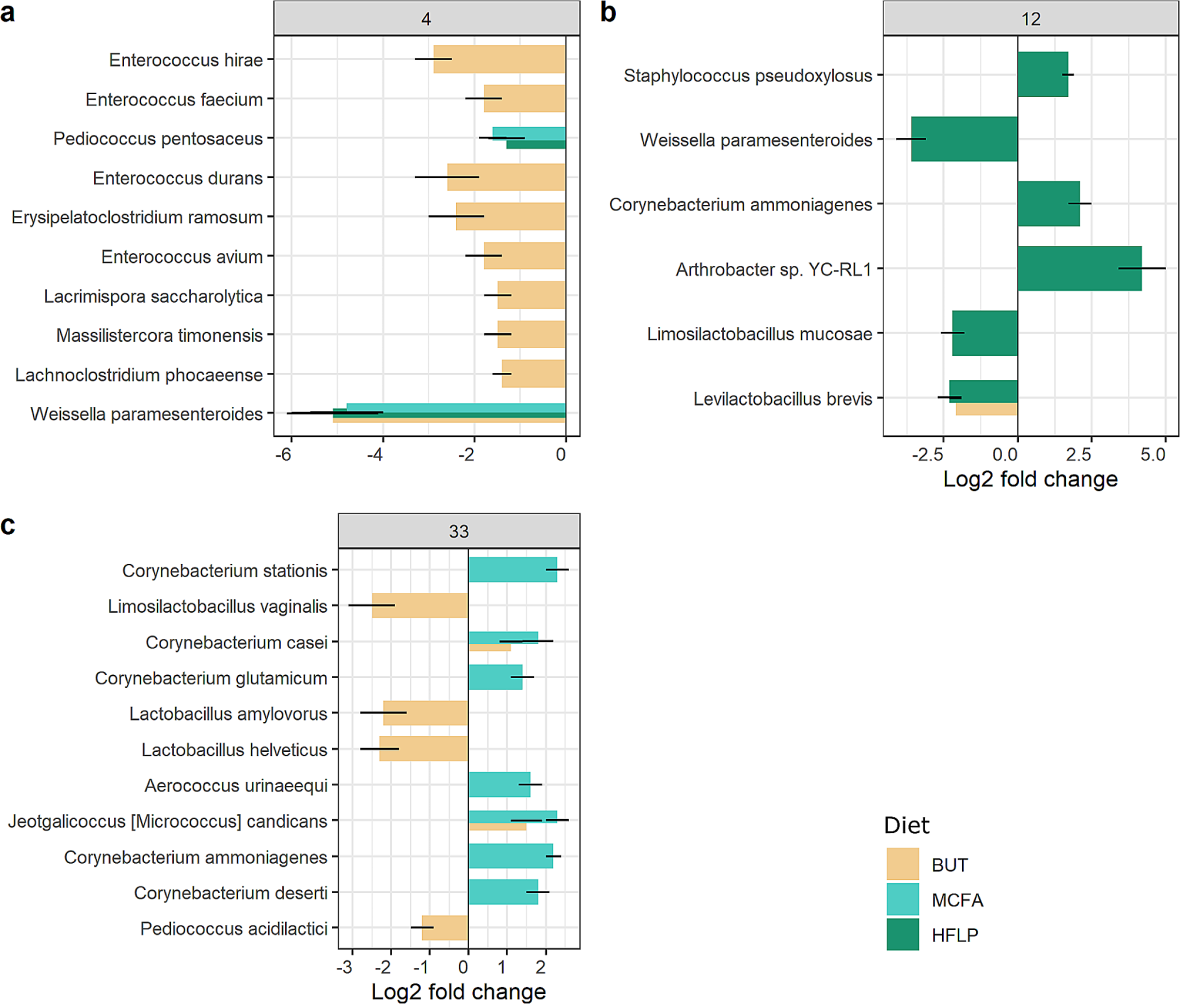
Differentially abundant bacteria in jejunal microbiota per diet group at 4, 12, 33 days post-hatch. Differential abundance analysis on BUT, MCFA and HFLP diet groups as a function of the control diet group at (**a**) 4 days post-hatch, (**b**) 12 days post-hatch and (**c**) 33 days post-hatch. Log2 fold change (l2fc) differences are visualized by bars and the standard error by error bars. Diet group are coloured (see legend). Differentially abundant bacteria are visualized (abundance > 0.001% ; *p*-value < 0.05; l2fc > |1|) and ordered from most abundant (top) to least abundant (bottom)

Analysis of sequencing data was performed in R version 4.0, Rstudio v2022.02.2 + 485 and functions of R packages phyloseq (version 1.4) and ggplot2 [82, 83]. The top 10 abundant bacteria in the jejunal microbiota were plotted by applying taxonomic agglomeration on species level (tax_glom, phyloseq package) while removing unassigned reads and extracting and plotting the ten most abundant bacteria using the aggregate function of microbiome utilities and plotting functions of the microbiome package [84, 85]. Bray-Curtis dissimilarity was used to evaluate difference in community structure on OTU level, using Hellinger-transformed abundances. Community composition was visualized with principal coordinates analyses (PCoA) of Bray-Curtis dissimilarity using functions of phyloseq and the microbiome packages [82, 85]. Permutational Multivariate Analysis of Variance (PERMANOVA) and tests on homogeneity of dispersion were employed using the adonis2 function (999 permutations, seed of 194,175) and betadisper function from the vegan package [86]. Principle Response Curve analysis was used to illustrate the trends for diet on the microbiome across different age groups [87]. Bacterial species were first filtered for at least 10% prevalence and 0.001% abundance, after which the *prc* function of the vegan package was applied with 999 Monte Carlo iterations [86]. The Shannon diversity and Pielou evenness index were calculated on OTU level by first applying rarefaction to an equal library size (720,000 reads, matching the sample with the lowest number of reads), using the rarefy_even_depth function of phyloseq (set.seed = 194,175, replace = FALSE) [82]. Consequently, the alpha diversity function of the microbiome package was applied [85]. Differential abundance analysis was performed by first applying an overall 10% prevalence and 0.001% abundance cut-off for bacterial species across all diet groups. ANCOM-BC version 1.6.0 was subsequently applied with standard settings, including Bonferroni correction for false discovery rate, batch correction for cage blocks and an alpha of 0.05 [88]. Structural zeros were included in the analysis (*struc_zero = TRUE*) and are indicated in tables s1-s9. A subsequent cut-off of 0.01% abundance per bacterial species per diet group and an absolute fold change cut-off of 2 were applied to generate the differential abundance plot (Fig. 4). The validation of detected bacterial species was performed by listing the reference genomes from all enterococcal species detected by kraken and downloading the corresponding RefSeq sequence from the NCBI database, filtering on full genomes and selecting the top hit when sorting by significance. Potential plasmids were excluded from the reference genomes and the resulting genomes were used to create a database using KMA version 1.4.3 and the index function with settings *-sparse TG*. Sequencing reads were subsequently aligned to this database using KMA and settings − *1t1*, *-ca*¸ *-apm p* and *-ef* [89].

### Electronic supplementary material

Below is the link to the electronic supplementary material.

Supplementary Material 1

Supplementary Material 2

Supplementary Material 3

Supplementary Material 4

## Data Availability

Sequencing files have been submitted into the Sequence Read Archive (SRA) at the NCBI under accession number PRJNA952340. The phyloseq object is available at 10.5281/zenodo.7744071.

## Declarations

### ARRIVE guidelines

All methods are reported in accordance with ARRIVE guidelines.

## Abbreviations

BUT: Butyrate
CON: Control
HFLP: High-fibre low-protein
MCFA: Medium chain fatty acids
MSS: Metagenomic shotgun sequencing
SCFA: Short-chain fatty acid
