# Supplementary material for: Diet-induced changes in the jejunal microbiota of developing broilers reduce the abundance of Enterococcus hirae and Enterococcus faecium
Source: BMC Genomics. 2024 Jun 23;25:627. doi: 10.1186/s12864-024-10496-8 (PMC11193906; doi:10.1186/s12864-024-10496-8)
Supplement: Supplementary file 4 — Supplementary Material 4 [file 12864_2024_10496_MOESM4_ESM.pdf]

Diet-induced changes in the jejunal microbiota of developing broilers reduce the abundance of *Enterococcus hirae* and *Enterococcus faecium*

Authors

Paul B. Stege[1*], Dirkjan Schokker[1], Frank Harders[1], Soumya K. Kar[2], Norbert Stockhofe[1], Vera Perricone[3], Johanna M. J. Rebel[1], Ingrid C. de Jong[2], Alex Bossers[1]

1. Wageningen Bioveterinary Research, Wageningen University and Research, Lelystad, Netherlands.

2. Wageningen Livestock Research, Wageningen University and Research, Wageningen, Netherlands.

3. Department of Veterinary Medicine and Animal Science, University of Milan, Italy.

Supplementary analysis

The observed effects in the abundance of *E*. *faecium*, *E*. *durans* and *E*. *hirae* were confirmed by realigning sequencing data of the control diet group 4 days post-hatch to the genome of all detected enterococcal species. This analysis was repeated for the bacteria *L*. *mucosae*, *L*. *vaginalis*, *L*. *brevis*, *L*. *amylovarus,* *L*. *helveticus*, *P*. *pentosaceus* and *W*. *paramesenteroides*.

L. *mucosae* and *L*. *brevis* were shown to decrease in abundance at 12 days post-hatch as a result of the HFLP diet compared to the control diet. At 12 days post-hatch butyrate supplementation results in a decreased abundance of *L*. *brevis*. Sequencing data of the control diet group 12 days post-hatch was mapped to the genome of all detected *Limosilactobacillus* and *Levilactobacillus* species (106.9M [SEM 4.5] reads per sample). This resulted in a high number of reads per sample mapping to *L. reuteri* (7.3M [SEM 1.6] reads, 82.1% [SEM 0.4] coverage , 403.1 [SEM 100.2] depth) while other detected *Limosilactobacillus* and *Levilactobacillus* species had a coverage below 70%. Due to the low number of reads and coverage, the differential abundance of *L*. *mucosae* and *L*. *brevis* at 12 days post-hatch could therefore not be confirmed.

L. *vaginalis*, *L*. *amylovarus* and *L*. *helveticus* were shown to decrease in abundance at 33 days post-hatch as a result of the BUT diet compared to the control diet. Sequencing data of the control diet group 33 days post-hatch was mapped to the genome of all detected *Limosilactobacillus* and *Lactobacillus* species (113.9M [SEM 35.0] reads per sample). This resulted in a high number of reads per sample mapping to *L. reuteri*, (1.8M [SEM 2.0] reads, 83.6% [SEM 5.9] coverage, 103.7 [SEM 128.5] depth), *L. vaginalis*, (0.4M [SEM 2.2] reads, 86.8% [SEM 10.9] coverage, 26.2 [SEM 140.5]

depth) and to *L*. *crispatus* (2.7M [SEM 14.4] reads, 87.1% [SEM 8.3] coverage, 154.1 [SEM 934.3] depth), while other detected *Limosilactobacillus* and *Lactobacillus* species had a coverage below 70%. Thus leading us to conclude that *L*. *vaginalis* is present in the jejunal microbiota of these broilers and affected by butyrate supplementation at 33 days post-hatch, while the differential abundance of *L*. *amylovarus* and *L*. *helveticus* could not be confirmed.

P. *pentosaceus* was shown to decrease in abundance at 4 days post-hatch as a result of MCFA and HFLP diet. *P*. *acidilactici* on the other hand, decreased in abundance at 33 days post hatch as a result of the BUT diet. When mapping the sequencing data of the control diet group at 4 and 33 days post-hatch to all detected *Pediococcus* species (105.1M [SEM 33.0] and 106.9M [SEM 4.5] reads per sample, 4 and 33 days post-hatch respectively). This resulted in a high number of reads per sample mapping to *P*. *acidilactici* 4 days post-hatch (1.3M [SEM 1.4] reads, 87.2% [SEM 1.0] coverage , 76.3 [SEM 92.5] depth), while other detected *Pediococcus* species at 4 or 33 days post-hatch had a coverage below 70%. Due to the low number of reads and coverage, the differential abundance of *P*. *pentosaceus* at 4 days post-hatch and *P*. *acidilactici* 33 days post-hatch could therefore not be confirmed.

Finally, *W*. *paramesenteroides* was shown to decrease in abundance at 4 days post hatch for the BUT, MCFA and HFLP diet and at 12 days post hatch for the HFLP diet. When mapping the sequencing data of the control diet group at 4 and 12 days post-hatch to all detected *Weisella* species (105.1M [SEM 33.0] and 106.9M [SEM 4.5] reads per sample, 4 and 12 days post-hatch respectively). The detected *Weisella* species at 4 or 12 days post-hatch had a coverage below 70% and therefore the

differential abundance of *W. paramesenteroides* at these timepoints cannot be confirmed.

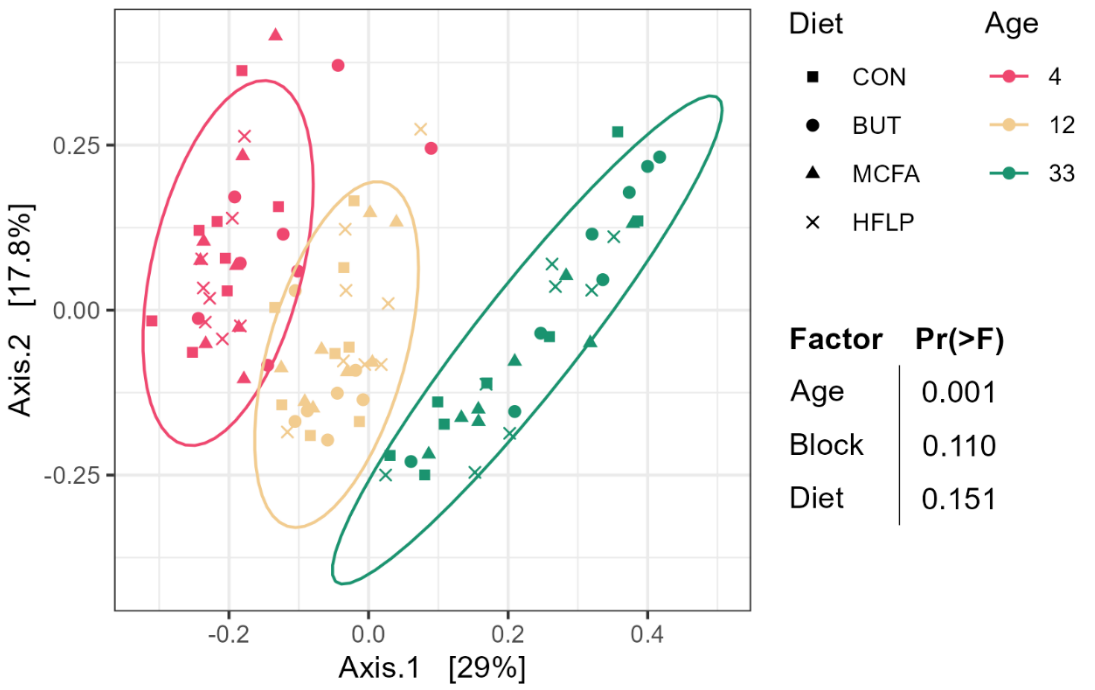

Figure s1 | Beta diversity of jejunal microbiota of diet groups and age groups. Beta-diversity of bacterial species, using principal coordinate analysis (PCoA) of Bray-Curtis dissimilarity on OTU level. Individual broilers and corresponding ellipses are coloured according to age group, while diet groups are marked by shapes (see legend). Permutational multivariate analysis of variance (PERMANOVA) and testing for homogeneity of multivariate dispersions both revealed significant differences between age groups (P=0.001).

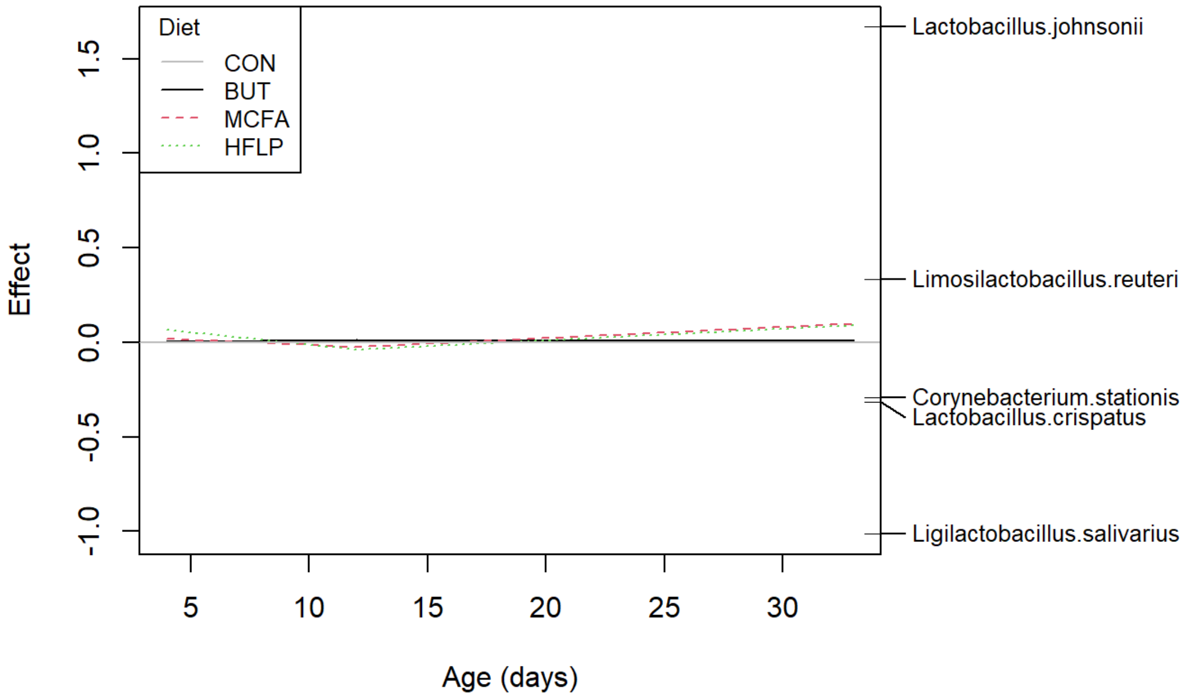

Figure s2 | Principle Response Curves of diet groups and age groups. Principle Response Curve analysis applied on the microbiome for diet groups across different age groups compared to the control group. The five species with the largest effect size are displayed and diet groups are coloured (see legend).

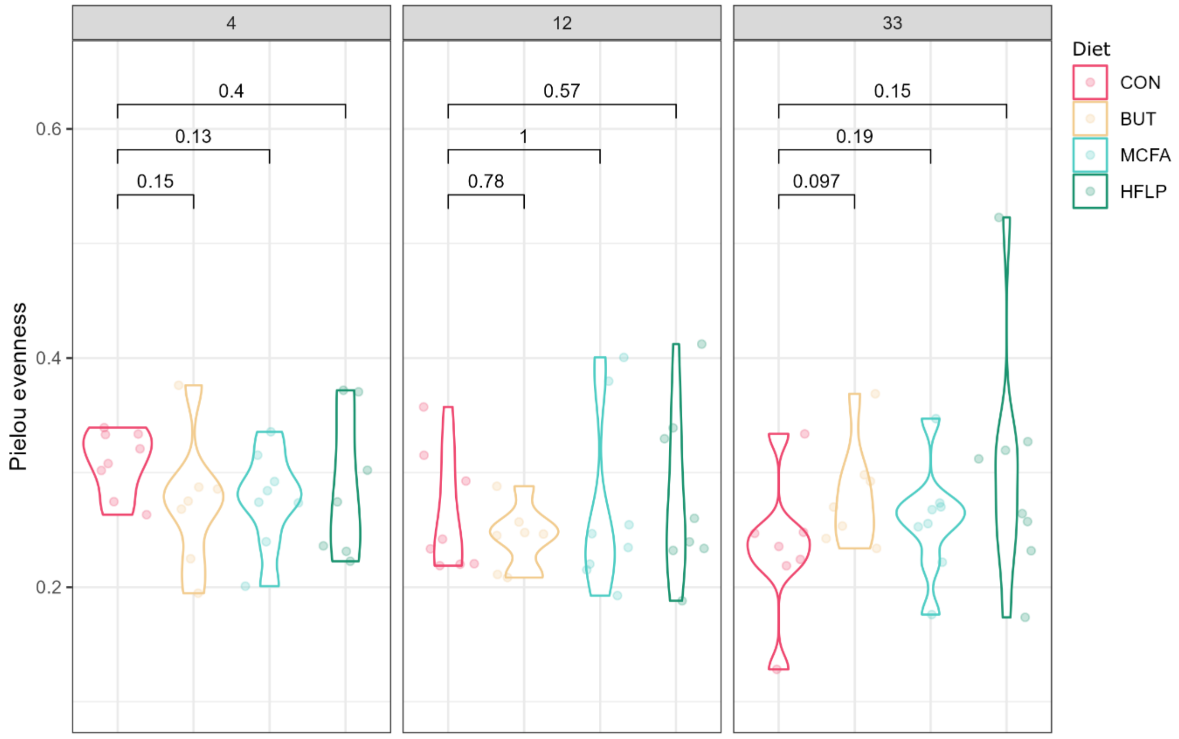

Figure s3 | Microbiota evenness per diet group. Alpha diversity per diet group expressed by Pielou evenness index on OTU level. Diet groups did not differ in terms of alpha diversity when compared with Wilcoxon rank-sum tests.
